# A comparison of face-to-face endotracheal intubation and standard intubation using Airtraq video laryngoscope in morbidly obese patients: A randomized controlled trial

**DOI:** 10.1097/MD.0000000000032046

**Published:** 2022-12-02

**Authors:** Justyna Nowak-Tim, Tomasz Gaszynski, Pawel Ratajczyk

**Affiliations:** a Department of Anaesthesiology and Intensive Therapy, Medical University of Lodz, Lodz, Poland.

**Keywords:** morbid obesity, airway management, general anesthesia, face-to-face intubation

## Abstract

Routine endotracheal intubation requires a patient in supine position with an operator standing behind the patient’s head. In case of a morbidly obese patient positioned in the recommended semi-recumbent position, an alternative method can be considered. Face-to-face intubation can be used both in patients in sitting as well as in prone position and when there is difficult access to the head. Evaluation of effectiveness and safety of face-to-face intubation in morbidly obese adult patients with body mass index over 40 kg m^−2^.

**Methods:**

The study was approved by the Local Ethics Committee and written informed consent from patients was obtained. We conducted a parallel randomized controlled trial with patients scheduled for elective sleeve gastrectomy. The trial was registered in ClinicalTrials with a number NCT04959149. Randomization and allocation to trial groups were carried out using the envelope method. The primary outcomes were the time of intubation and the first pass success of endotracheal intubation.

**Results:**

76 patients (routine intubation n = 36, face-to-face intubation n = 40) were included in the study with no dropouts. The intubation success rates were 82.5% versus 100%, mean intubation time was 17.1 ± 18 seconds versus 29 ± 11 seconds and the need for additional maneuvers (backward, upward, rightward pressure or flexing the neck) was 15% versus 19.5%, in face-to-face and routine intubation, respectively. No injuries to teeth or mucosa have been reported. There were no incidents of desaturation below 92% or other complications associated with intubation.

**Conclusion:**

Face-to-face intubation is shorter than the routine intubation in obese patients. This method may be an alternative to standard intubation in case of airway management in morbidly obese patients in semi-erect position; however, it requires both training and practice.

## 1. Introduction

**Figure 1.**
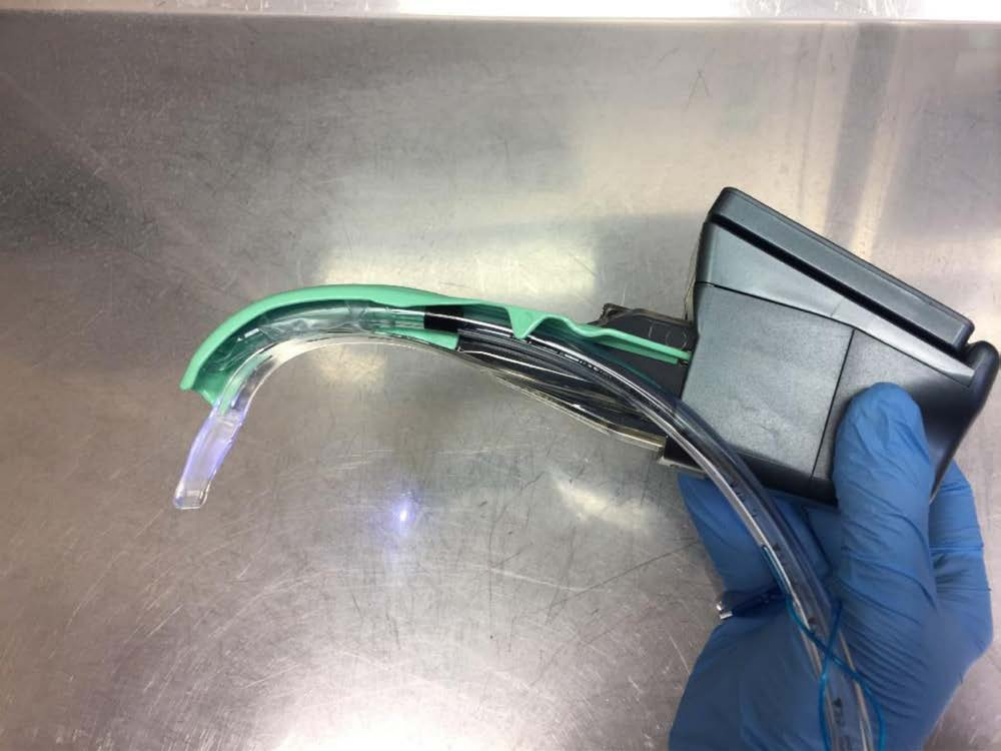
Airtraq video laryngoscope with a channel for endotracheal tube.

For standard endotracheal intubation, the patient is positioned in supine position, with the anesthesiologist standing behind his/her head and with adequate access to the head and neck of the patient. However, there are several situations where routine intubation is very difficult or even impossible. In immobilized trauma victims with limited access to the head, suspected cervical spine injury or in a patient in sitting position (e.g. spondyloarthritis), the intubation efforts performed by a person standing in front of a patient might be the only option for successful airway management.^[1–3]^ In case of general anesthesia in morbidly obese patients with body mass index over 40 kg m^−2^, the face-to-face method may be considered as the patient is placed in head-elevated laryngoscopy position with the upper body elevated. Such an approach is recommended in this group of patients.^[4,5]^ Postural change from supine to semi-erect position decreases the risk of airway obstruction caused by the collapse of pharyngeal soft tissues in patients undergoing general anesthesia and muscle relaxation in those suffering from obstructive sleep apnea.^[6,7]^ The face mask ventilation in a morbidly obese patient in supine position might be complicated due to the increased amount of fat tissue in the cheeks and palate, tonsils hypertrophy, larynx relocation, and limited mouth opening.^[8]^ Moreover, manipulations with a laryngoscope are restricted because of small space in the patient’s mouth, decreased neck mobility and elevation of the chest. Intubation failures may reach up to 13% in this group of patients.^[8]^ These are the reasons why postural change in morbidly obese patients might be crucial to ensure optimal ventilation and intubation conditions. These can be improved by the use of optical or video laryngoscopes.^[8]^ The Airtraq video laryngoscope allows an increase of the effectiveness of the glottic opening visualization and is associated with a reduction in the need of additional maneuvers during intubation efforts.^[1,9]^ This device can be equipped with an endotracheal tube guiding channel—the channeled blade (Fig. 1). There is also a nonchanneled blade version available. This video laryngoscope can be used with a screen attached to the device or can be connected to a separate monitor. The additional advantage is the possibility to record the video for medical and teaching purposes. It is available in several sizes and can be used in adults and children. The aim of this study is to compare the effectiveness of face-to-face endotracheal intubation and routine intubation, both with the use of the Airtraq video laryngoscope (Prodol, Meditec SA, Vizcaya, Spain). Our hypothesis is that face-to-face intubation is more effective than the standard method in obese patients.

## 2. Methods

The study was approved by the Local Ethics Committee No. RNN/62/20/KE and written informed consent was obtained from all participants. We conducted a parallel randomized controlled trial in 76 patients aged 21–55 years, with body mass index over 40 kg/m^2^. The trial was registered in ClinicalTrials with a number NCT04959149. The study was designed to assess the noninferiority of face-to-face intubation in comparison to standard endotracheal intubation. Randomization and allocation to trial group were carried out using the envelope method. Forty face-to-face endotracheal intubations (face-to-face group) and 36 routine intubations (standard intubation group) were performed using the Airtraq video laryngoscope for elective sleeve gastrectomy under general anesthesia.

**Figure 2.**
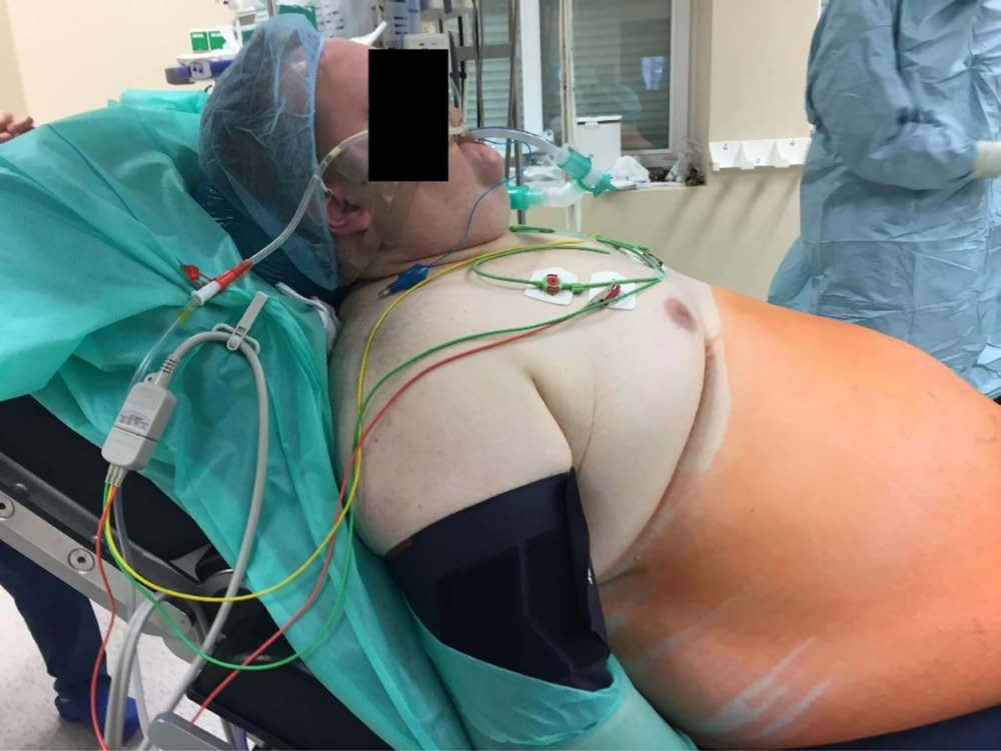
Upper body elevation in a bariatric patient.

**Figure 3.**
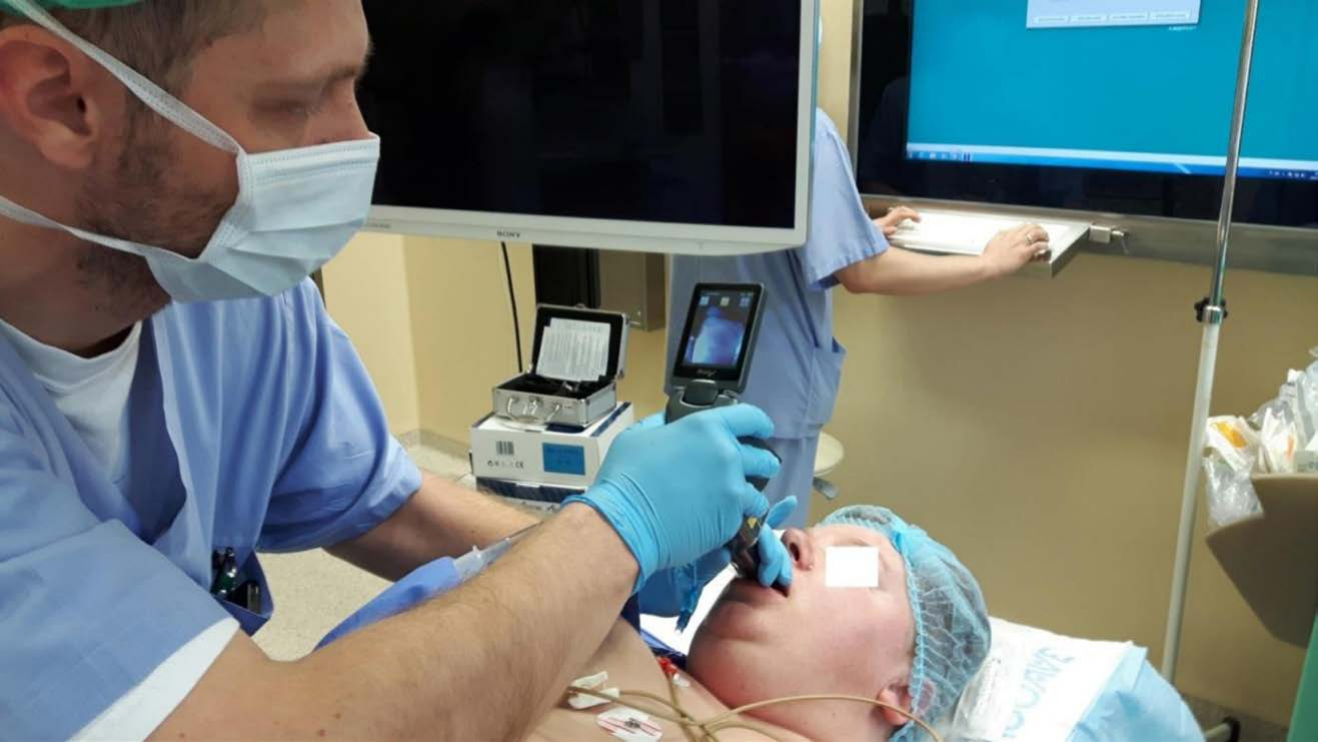
Intubation in face-to-face technique.

The inclusion criteria were body mass index over 40 kg m^−2^, both sexes, surgery requiring general anesthesia with endotracheal intubation. The exclusion criteria were lack of consent, emergency surgery, rapid sequence induction, expected difficult intubation, recent upper respiratory tract infection (i.e. within 14 d), an airway tumor or previous thyroid gland surgery. Data collection period was March 2020—September 2020. We assessed the following parameters: intubation time, success rate, and necessity of using additional maneuvers. The primary outcomes were time to intubation and first pass success rate. Patients admitted to hospital for elective bariatric procedures under general anesthesia were included into the study. All patients were positioned with the upper body elevated approximately 30 degrees (Fig. 2). The standard anesthesia monitoring consisted of electrocardiogram, pulse oximetry, noninvasive blood pressure measurement, end-tidal carbon dioxide, and train-of-four monitoring. Patients were positioned in head-elevated laryngoscopy position position for routine intubation. All patients were preoxygenated for 5 minutes. with 100% oxygen using facemask and continuous positive airway pressure. The induction of general anesthesia was performed by the intravenous administration of 1–2 µg kg^−1^ of fentanyl and 2–2.5 mg kg^−1^ propofol. A dose of 0.6 mg kg^−1^ rocuronium was administrated once the face mask ventilation was confirmed to be possible. The intubation attempt commenced once an adequate muscle relaxation has been achieved as per train-of-four monitoring. The Airtraq laryngoscopes with channeled blades were used. In the face-to-face method the anesthesiologist was standing in front of a patient (Fig. 3). In the standard intubation group, the anesthesiologist was standing behind the patient’s head. Afterwards, general anesthesia was conducted with desflurane and a mixture of oxygen and air. In the face-to-face group, the intubating anesthetist was standing in front and on the right side of a patient. A 7.5 internal diameter endotracheal tube for females and a 8.0 ID endotracheal tube for males were used. In both groups, the entry to the larynx was visualized on the screen attached to the device. The Airtraq manufacturer’s instructions for users were followed.^[10]^ Our primary outcome was the time of intubation. This parameter was measured from the patient’s mouth opening to the confirmation of correct placement of the tube confirmed by EtCO2 monitoring by assistant. Secondary outcomes were: the need of additional maneuvers (backward, upward, rightward pressure or flexing the neck), esophageal intubation, hypoxaemic incidents (a drop in saturation < 92%), mucosal, or teeth injuries were noted. The presence of sore throat or dysphagia were assessed during a 2-hour stay in the post-anesthesia care unit by an independent observer. In case of prolonged time of face-to-face intubation (>120 s) or 2 unsuccessful attempts, patients were intubated in a standard way with the same video laryngoscope. We analyzed data of 76 patients in Statistica (13.3) TIBICO Software Inc. We used Student *t* test to analyze normally distributed continuous variables and the Mann–Whitney *U* test to analyze continuous variables with a distribution other than normal. Spearman’s rank correlation test was used to compare the correlation between continuous variables. The Yates’s Chi-squared test was used for the comparison of dichotomous variables.

The sample size was estimated with the following assumptions:

- The difference in mean intubation times between the methods (Δμ) is 10 s which results from our previous observations,
- Standard deviation (σ): 10 seconds,
- The size of the groups is equal (N1 = N2),
- Significance level (ɑ): 0.05,
- Test power: 90%,
- Intubation times are normally distributed,
- Student’s *t* test for independent samples.

Based on the above criteria 50 patients were required for the study.

## 3. Results

There were no significant differences between analyzed groups regarding demographic features and airway characteristics (Table 1). The correlation between demographic features is presented in Table 2.

**Table 1:** Patient demographic features and airway characteristics.

	Face-to-face (n = 40)	Typical (n = 36)	*P* value
Age	40.50 (8.45)	39.44 (11.62)	.650
Weight	128.38 (18.22)	124.17 (18.64)	.323
Height	170.13 (7.72)	168.89 (7.98)	.495
BMI	44.30 (5.14)	43.43 (5.02)	.459
Mallampati 1/2/3/4	0/20/17/3	0/18/16/2	

For the normally distributed variables (age, weight, height, BMI body mass index), the mean and standard deviation are presented. For variables with a distribution other than normal (Mallampati score), the median and value (min–max) are presented. Student’s *t* test was used to compare variables with a normal distribution, the Mann–Whitney *U* test was used for variables with a distribution other than the normal.

BMI = body mass index.

**Table 2:** The correlation between demographic features and Mallampati score.

	Weight	Height	BMI	Mallampati score	Intubation time
Age	0.12	0.12	0.07	−0.22	0.01
	Weight	0.66	0.73	0.28	−0.04
		Height	0.02	0.17	0.06
			BMI	0.23	−0.09
				Mallampati Score	0.01

The graph shows the Spearman’s rank correlation coefficient.

BMI = body mass index.

Statistically significant results (*P* < .05) are marked in red.

To compare intubation time in the 2 groups the Mann–Whitney *U* test was used. It revealed that time of intubation in face-to-face group was shorter than in the standard method: median 10 versus 28 seconds, respectively. The mean values were 17.1 ± 18 versus 29 ± 11 seconds in face-to-face and standard method, respectively (Table 3). However, success rate in the standard method was higher than in face-to-face approach (100% vs 82.5%, respectively) with *P* value of 0.025 in Chi-squared test (Table 4). The rate of additional maneuvers was 15 versus 19.5 % in face-to-face and routine intubation, respectively.

**Table 3:** Comparison of intubation time.

	Face-to-face (n = 40)	Typical (n = 36)	*P* value
Intubation time (s)	10 (6; 120)	28 (14; 62)	.000

The table shows the median and the value (min–max). The Mann–Whitney U test was used to compare the variables.

**Table 4:** Comparison of successful rate.

	Face-to-face (n = 40)	Typical (n = 36)	*P* value
Successful intubations (number and percentage) was calculated using the χ^2^ test with Yates correction	33 (82.5%)	36 (100%)	.0252

In 7 cases of face-to-face intubation, we decided to intubate with a standard approach because of prolonged (>120 s) intubation time. These cases were treated as unsuccessful intubation with a duration time of 120 seconds and included in our calculation.

The baseline vital parameters during the whole procedure remained stable and within normal ranges. There were no complications such as teeth damage, esophageal intubation, aspiration, desaturation, or bleeding recorded. There were no reports from postanesthesia care unit regarding sore throat or dysphagia. Written consent for photos publication were obtained from all patients.

## 4. Discussion

Face-to-face intubation is also known as the ’tomahawk’ or “pickaxe” method. It can be used for patients entrapped in a vehicle^[2]^ or in patients with spondyloarthritis. Morbidly obese patients may benefit from face-to-face intubation because the head elevated position or semi-erected position allows better oxygenation during induction of anesthesia.^[6]^ However, this method needs experience and can be used after proper training, for example in a manikin model.^[11]^

Our study demonstrated that face-to-face intubation is shorter than the routine one. These results are in opposition to the outcomes of some authors who found out that intubation times in a standard and face-to-face method are comparable.^[1,11,12]^ There are some authors, who describe a possibility of using Macintosh laryngoscope for a face-to-face intubation^[13–15]^; however, it seems that a video laryngoscope is more suitable for this technique, achieving higher effectiveness, shorter intubation time and is more user-friendly for an anesthesiologist.^[16,17]^ Moreover, video laryngoscopy does not require extensive cervical spine movements, which might be essential in morbidly obese group of patients due to restricted neck mobility.^[18]^ Some authors report that face-to-face intubation can be successfully performed by one person with no need of an assistant.^[19,20]^ An anesthesiologist (standing in front and on the right side of the patient) can hold a video laryngoscope with his/her right hand and insert the tube with the left one.^[21,22]^ However, in a study by Shah et al, the authors revealed that operators experienced with videolaryngoscope perform better face-to-face intubation when holding the video laryngosope in their left hand.^[23]^ It is optional to introduce the device with the left hand (like in a standard technique) and after obtaining a satisfactory view of the laryngeal inlet, relocate a video laryngoscope to the right hand and insert intubation tube with the left one. Independently from the method, a video laryngoscope is a better choice than a standard Macintosh device. However, we have to highlight that in the face-to-face method the picture on the screen of a video laryngoscope is reversed upside down and it might require some training to become familiar with this technique and even experienced anesthesiologist might find it difficult. The skill of face-to-face intubation enables airway management in a patient in an unusual position not only in bariatric procedures.^[3]^ Another point to discuss is using a video laryngoscope with a channel (a channeled blade) which might be a facilitation of an intubation technique. It is difficult to define how important using the channeled blade was regarding the face-to-face method for results of the study. It may be possible to use intubation stylet placed in the tube with a video laryngoscope without the channeled blade for face-to-face intubation. According to the literature, the frontal approach can also be performed using non-channeled devices.^[1,6,11]^

There is no other paper on evaluation of face-to-face intubation in morbidly obese patients. When comparing the results of the trials performed on non-obese patients, Arslan et al received a higher success rate (100%) using the Airtraq in face-to-face technique than in our study (82.5%).^[1]^ The time to intubation was also shorter (8 s) compared to our results (17.1 s). This can be explained by difficulties in obese patients compared to the non-obese. Obesity can be associated with longer intubation time because of specific intubation conditions in such patients.^[8]^ Julliard et al performed a study on a cadaver model comparing face-to-face intubation and a standard approach for intubation using the C-Mac video laryngoscope.^[24]^ They achieved 19 and 20 seconds for both methods, respectively, which is similar to our results in morbidly obese patients.

Amathieu et al performed a manikin study in simulation settings with a scenario of an entrapped patient with face-to-face intubation using the AirTraq video laryngoscope.^[21]^ Their results were in opposition to our findings regarding the success rate in face-to-face intubation: 100% versus 82.5%, respectively. The time of intubation was also shorter both for a standard and face-to-face intubation technique than in our study: 8 and 14 seconds versus 29 and 17.1 seconds, respectively. The difference in the success rate is difficult to explain. The possible explanation is that the face-to-face technique is a more requiring technique especially in the morbidly obese. Gaszyńska et al performed a similar study on a manikin model simulating face-to-face intubation of an entrapped patient using different intubation devices.^[25]^ When comparing the time of intubation by paramedic students using the AirTraq for a face-to-face intubation technique in a manikin positioned under the table, Gaszyńska achieved similar results to the standard technique: 25.2 versus 22.9 seconds, respectively. This is similar to our results regarding time of intubation. In a study of Venezia et al on intubation in face-to-face approach performed by novice operators in a manikin model, the authors found that such a technique was more effective than the routine intubation.^[26]^ They achieved 100% success in face-to-face intubation compared to 91% in a standard technique. This is contrary to the results of our study, where the success rate for face-to-face intubation was 82.5% and for the standard technique of intubation was 100%.

The main limitation of our study is that intubation attempts in both groups were performed by different operators with different experience in intubation. Therefore, is it difficult to assess whether the face-to-face method would be as simple as the standard one for the same operator intubating the same patient. The main strength of this study is that the research is randomized, prospective and is carried out in a group of obese patients with expected difficult intubation and that is why it has considerable clinical importance.

## 5. Conclusion

Face-to-face intubation is shorter than routine intubation but is associated with more unsuccessful attempts. Its use was not related to an increased risk of the following complications: desaturation, dysphagia, sore throat and tissue or teeth damage.

## Acknowledgments

Authors thank dr Dawid Aleksandrowicz MD PhD (London North West Healthcare NHS Trust) for reviewing and correcting manuscript.

## Author contributions

**Conceptualization:** Tomasz Gaszynski.

**Data curation:** Justyna Nowak-Tim.

**Formal analysis:** Justyna Nowak-Tim.

**Investigation:** Justyna Nowak-Tim.

**Methodology:** Tomasz Gaszynski.

**Supervision:** Tomasz Gaszynski.

**Writing—review and editing:** Pawel Ratajczyk.
